# Lack of Transmission of Vaccinia Virus

**DOI:** 10.3201/eid1204.051373

**Published:** 2006-04

**Authors:** James H. Stark, Sharon E. Frey, Paul S. Blum, Thomas P. Monath

**Affiliations:** * Acambis Inc., Cambridge, Massachusetts, USA;; † St. Louis University School of Medicine, St. Louis, Missouri, USA

**Keywords:** Vaccinia, smallpox, transmission, vaccine, bandage, letter

**To the Editor:** Recently, the US government completed a targeted vaccination strategy limited to healthcare workers, first responders, and the military because of concern that variola virus, the etiologic agent of smallpox, might be used as a biowarfare agent (*1*). A concern in such programs is the potential for unintended spread of the vaccine virus (vaccinia) from the primary vaccinee to contacts who may be at the greatest risk of having adverse reactions resulting from secondary transmission (*2**,**3*).

Contact spread of the live attenuated vaccinia virus is considered the predominant method of secondary transmission. The conventional methods of preventing a secondary transmission event in the household of a smallpox vaccine recipient include the use of bandages and long sleeves to limit direct contact with the lesion and immediate hand-washing when contact occurs (*4*).

Several recent reports have measured the presence of vaccinia virus on the dressings or hands of vaccinated persons; however, the recovery of vaccinia virus in the environment has not been evaluated after vaccination in a controlled setting (*5**–**7*). We present the first reported attempt to recover live vaccinia virus from the homes of recently vaccinated persons. This study was approved by the St. Louis University Institutional Review Board. We hypothesized that live vaccinia virus shed from the skin reaction could not be recovered in the natural environment, and as a result, constitutes a limited risk for contact transmission.

Three hundred eighty-seven environmental swab samples were collected on 3 different study days from 43 persons (mean age 24 years) with major cutaneous reactions. Persons who participated in this study were selected from a randomized, double-blind, single-center study that compared the safety, tolerability, and immunogenicity of 3 smallpox vaccines (*8**,**9*). Following vaccination and after each study visit, the vaccination site was covered with an OpSite Post-Op dressing (Smith and Nephew, Massilon, OH, USA). On postvaccination days 7, 10, and 15, a sterile Calgiswab type 2 applicator (Harwood Products Co., Guilford, ME, USA), moistened in sterile water, was rotated over the linen from the study participant's bed (approximate location of sleeping area), the middle of his or her bath towel, and the inside area of a shirt sleeve adjacent to the vaccination bandage (before laundering). These sampling areas were chosen on the basis of the likelihood of exposure to the semipermeable bandage and the potential for another person to come in contact with the vaccinia virus in these areas. An additional 129 samples from the palm of the study participant's hand used to take the environmental samples were taken to serve as a control mechanism.

After sampling, the tip of the swab was stored in a 15-mL conical tube containing 3 mL multimicrobe transport media (Remel, Lenexa, KS, USA). The 15-mL conical tubes were returned to the clinic in a cooler on cold packs the same day. Recovery of vaccinia virus was determined by infectivity assay. Samples were tested for infectious vaccinia virus by inoculation of fluid cultures of Vero cells grown in 12-well plates. A sample was defined as positive if cytopathic effects were observed (*10*).

Concurrent with the environmental sampling, the lesion and the outside of the bandage covering the inoculation site for each study participant were swabbed with a Calgiswab Type 2 sterile applicator, and the samples were analyzed by infectivity assay. These samples served as a positive control, indicating that the method used to sample the environment was appropriate and sensitive.

All 516 environmental samples from designated sampling areas in the homes of recently vaccinated vaccinia-naive persons were negative for live virus as determined by plaque infectivity assay (Table). Only 1 (0.78%) of 129 dressing samples tested on day 7 had measurable titers of vaccinia.

**Table:** Sampling outcomes of 43 study participants who received 1 of 3 smallpox vaccines

Variable	All participants (N = 43)	Vaccine group		
		ACAM1000 (n = 14)	ACAM2000 (n = 15)	Dryvax (n = 14)
Mean ± SD titer from lesion*†‡				
Day 7	3.84 ± 0.98	3.77 ± 1.15	3.85 ± 1.06	3.91 ± 0.74
Day 10	3.75 ± 0.89	3.62 ± 0.54	3.58 ± 1.21	4.07 ± 0.74
Day 15	4.19 ± 1.40	4.08 ± 1.24	3.67 ± 1.57	4.86 ± 1.18
Mean ± SD titer from dressing§				
Day 7	0.63 ± 0.85	0.9 ± 1.50	0	0
Day 10	0	0	0	0
Day 15	0	0	0	0

*Viral shedding results reported as <X were converted to X/2 before calculating. †Titers are reported as log_10_ PFU/mL. ‡There were no significant differences among the groups (p>0.05). §Limit of detection was 1.0 log_10_ PFU/mL.

Contact with live vaccinia virus from the lesion at the site of vaccination is the underlying cause of secondary transmission. Common mechanisms for transmission include contact with contaminated bandages and intimate sexual contact. Recent studies have compared a variety of bandages used to cover the vaccination site to determine which class of bandage provides the greatest protection against disseminated virus. Talbot et al. observed that <1% (N = 918) of dressing samples were positive for vaccinia (an initial semipermeable OpSite Post-Op dressing and an outer semipermeable Tegaderm bandage) (*5*). In a single-blind randomized trial design, Waibel et al. compared the presence of vaccinia virus on the external surface of 3 different types of bandages and noted that the semipermeable membrane with gauze had the smallest proportion of recoverable virus compared with the groups that used a Band-Aid or double gauze with adherent tape (*6*). Despite the difference in types of bandages from these studies, the results were remarkably consistent with regard to the limited dissemination of vaccinia virus outside the dressing. In concordance with our results, the semipermeable bandage provided significant protection from exposure to the virus on the outside of the bandage.

This study presented many challenges regarding the sampling and collection of specimens. Collection times, sampling technique, and environmental conditions may have contributed to the absence of viral recovery. In addition, we chose to measure live vaccinia virus as opposed to noninfectious viral genomes (by polymerase chain reaction) because we were concerned with transmission and infectivity. If we had chosen to measure noninfectious viral genomes, a positive outcome may have been likely. However, from a public health standpoint, such information would have been less valuable. Further studies will need to examine the viability of vaccinia virus in the environment to evaluate the possibility of contact transmission.
